# Knowledge about Fibromyalgia in Fibromyalgia Patients and Its Relation to HRQoL and Physical Activity

**DOI:** 10.3390/biology10070673

**Published:** 2021-07-16

**Authors:** María Mendoza-Muñoz, Jesús Morenas-Martín, Miguel Rodal, Judith García-Matador, Miguel Ángel García-Gordillo, José Ignacio Calzada-Rodríguez

**Affiliations:** 1 Health, Economy, Motricity and Education Research Group (HEME), Faculty of Sport Sciences, University of Extremadura, 10003 Caceres, Spain; mamendozam@unex.es (M.M.-M.); judgm@hotmail.com (J.G.-M.); jocalzada@alumnos.unex.es (J.I.C.-R.); 2 Motor Control Research Group, Faculty of Sport Sciences, University of Extremadura, 10003 Caceres, Spain; 3 BioErgon Research Group, Faculty of Sport Sciences, University of Extremadura, 10003 Caceres, Spain; mrodal@unex.es; 4 Universidad Autónoma de Chile, Talca 3467987, Chile; miguel.garcia@uautonoma.cl

**Keywords:** HRQoL, knowledge, physical activity, rheumatic diseases, women

## Abstract

**Simple Summary:**

Fibromyalgia (FM) affects 2.40% of the Spanish population and its most widespread treatment has been the combination of patient education, pain coping strategies and exercise, in this sense, with respect to patient education, few studies have tried to see the relationship between education in FM with an improvement in FM. Therefore, the aim of this study was to know the level of knowledge about FM among patients in Extremadura, to explore the relationship between knowledge of FM and health-related quality of life (HRQoL), and to analyze the relationship between knowledge of physical activity in FM and the practice of physical activity. For this purpose, 121 women with a mean age of 55.06 years were evaluated. It was found that 10% of these women had low knowledge of FM, 49% medium and 41% high. It was also found that the level of knowledge of physical activity was only weakly related to HRQOL and body pain. Thus, it was concluded that the level of knowledge about FM of patients in Extremadura was medium-high and that there was a weak relationship between knowledge about physical activity in FM and HRQOL. However, no relationship was found between knowledge of physical activity in FM and the practice of physical activity.

**Abstract:**

*Introduction*: Fibromyalgia (FM) affects 2.40% of the Spanish population. The most widespread treatment has been the combination of patient education, pain coping strategies and exercise. With regard to patient education, there are few previous studies on the efficacy of relating FM education in isolation with an improvement in FM, although there are some studies that report that health education programs could modify the perception of quality of life and improve pain. *Objectives*: the aim was to find out the level of knowledge about FM among patients in Extremadura, to explore the relationship between knowledge of FM and Health-Related Quality of Life (HRQoL) and to analyze the relationship between knowledge of physical activity in FM and the practice of physical activity. *Methods*: A single-measure cross-sectional study was carried out with 121 women with a mean age of 55.06 (±9.93) years. The following questionnaires were used: Fibromyalgia Knowledge Questionnaire (FKQ); SF12v2 (Short-Form Health Survey); and EURO-QOL-5D-5L (EQ-5D-5L). *Results*: regarding the level of knowledge of the participants about FM, it was found that 10% had a low knowledge, 49% medium and 41% high. In relation to the associations between the level of knowledge and HRQoL, a weak correlation between EQ-5D-5L and the FKQ in the domain of physical activity (r = 0.243) were found. *Conclusions*: it can be concluded that the level of knowledge about FM of the patients from Extremadura was medium-high and that there is a direct weak relationship between knowledge about physical activity in FM and HRQoL. However, no association was found between knowledge of physical activity in FM and the practice of physical activity.

## 1. Introduction

Fibromyalgia (FM) was recognised as a disease in the Copenhagen Declaration by the World Health Organisation in 1992 [1]. It is the second most frequent condition among rheumatic diseases [2] and it can be considered as a form of non-articular rheumatism characterised by chronic diffuse musculoskeletal pain, together with the presence of multiple pressure sore spots [3], implying a great impact on the physical, psychological and social well-being of the patient [2]. 

FM affects 2.7% of the world population, being more prevalent in 50-year-old or older women [4]. In Spain, FM affects of 2.40% of the population [5], being associated with females aged between 40 and 59 years [6] who suffer from diffuse musculoskeletal pain, aches or stiffness associated with fatigue, anxiety, poor sleep, headaches, irritable bowel syndrome, subjective swelling in arthritic and periarticular areas, and numbness [7]. In this line, it has been shown that the factors that can reduce health and quality of life are widespread pain and tenderness, cognitive problems, non-recovery sleep, fatigue, depression, anxiety, poor physical fitness, stiffness and mobility or balance problems [8].

Currently, there is no cure for this pathology, but by applying different multidisciplinary treatments, improvements in quality of life at the physical, psychological and social levels can be achieved [9,10]. Therefore, treatment depends not only on the use of pharmacologic therapy, but also on the implementation of intervention programmes, often of a cognitive-behavioural nature and physical exercise [11,12,13].

Previous studies on the impact of FM on quality of life suggest that the most effective treatment would be a combination of patient education, pain coping strategies and aerobic exercise [14]. Specifically, related to patient education, previous studies on the efficacy of linking information/education on FM in isolation to a noticeable improvement in FM can be hardly found. Nevertheless, Koca, et al. [15] state that patients’ knowledge about FM could contributes to the control of disease and other studies have reported that health education programmes could modify the perception of quality of life and improve pain relief, as well as decrease dependence on health services [16], or that education in pain physiology seems to improve health status and long-term endogenous pain inhibition [17]. However, in most studies, information/education is approached from a multidisciplinary point of view, i.e., it is associated with other types of treatment such as physical activity, with very beneficial results [18,19,20].

In relation to the content of information/education, at the societal level, the increased interest in FM has contributed to confusion with claims lacking scientific rigour, as many questions have been raised about people with FM and many media have tried to answer them. Information should be direct, objective and in accordance with the scientific knowledge that exists about FM, since improving knowledge about FM can be useful for creating a social bond between people affected by this disease and health professionals, family members, friends and/or work colleagues, thereby facilitating their adaptation to the difficulties that the disease causes in their daily lives. 

Therefore, the aim was to find out the level of knowledge about FM among patients in Extremadura, as well as to explore the relationship between knowledge of FM and Health-Related Quality of Life (HRQoL) and, more specifically, to analyse the relationship between knowledge about physical activity in FM and the practice of physical activity.

## 2. Materials and Methods

### 2.1. Study Design 

A single measurement cross-sectional study was conducted.

### 2.2. Ethical Approval

Ethical approval was provided by the Bioethics and Biosafety Committee of the University of Extremadura (approval number: 51/2013).

### 2.3. Sample Calculation

One hundred and thirteen participants were needed to reach a power of 80% to detect a difference of 0.30 between the null hypothesis correlation of 0.29 (very low or close to zero association) and the alternative hypothesis correlation of 0.60 (high association) [21]. The significance level was set at alpha equal to 0.05. Futhermore, about ten percent more were recruited to meet this estimate

### 2.4. Participants

The total sample consisted of 121 women with a mean age of 55.06 (±9.93) years. Participants were recruited along one month from different associations of patients affected by FM syndrome (Mérida, Don Benito, Valencia de Alcántara, Alburquerque, Toledo and Almansa) and the “Manuel Encinas” Health Centre in Cáceres by the professionals working there. The inclusion criteria followed were: to be of legal age, to have a diagnosis of FM by a rheumatologist according the American College of Rheumatology criteria [8], not to present any disease affecting the understanding of the test and to sign the informed consent form.

### 2.5. Procedure, Material and Measurement

In this study, several instruments were used to assess knowledge about both FM and HRQoL, as well as to obtain characterisation data on the participants (age, level of education, years of diagnosis of the disease, distance spent walking per week, hours spent doing physical activity per week, etc.). The questionnaires were sent to the participants both by e-mail and by post. These were completed along one week in the centres mentioned above, with the help of a trained professional who got in contact with the research team. Finally, this material was sent to the research team together with the participants’ informed consent. The instruments used were:

Fibromyalgia Knowledge Questionnaire (FKQ) [22]. This questionnaire assesses the level of knowledge about FM. It is composed of 18 items, divided into four domains (general knowledge about the disease; knowledge about treatment, medication and possible side effects; knowledge about physical activity; and knowledge about day-to-day activities in relation to energy used or how best to save energy), with the total score of the questionnaire reaching 26 points (Cronbach’s alpha > 0.69).

Short-Form Health Survey (SF12v2) [23]. It is a generic measure of HRQoL, which is an abbreviated form of the SF-36. This instrument can be self-administered or completed through an interview. It contains 12 items addressing eight domains (physical functioning, physical role, body pain, general health, vitality, social function, emotional role, and mental health) and two summary scores related to physical health and mental health (Cronbach’s alpha > 0.70). With these dimensions, and by applying predetermined algorithms, the two summary scores are created: the physical health index (PHI) and the mental health index (MHI) [24].

EURO-QOL-5D-5L (EQ-5D-5L) [25]. This instrument was developed by the EuroQol Group with the aim of improving the previous version (EQ-5D-3L) [25]. The EQ-5D-5L describes the current health status of individuals across five dimensions (mobility, self-care, daily activities, pain/discomfort and anxiety/depression) (ICC: 0.69 to 0.94) [26]. Each of the dimensions has five possible responses with 1 being the best possible health status and 5 the worst. In addition, this questionnaire includes a visual analogue scale, which evaluates from 0 to 100 the health status of the person on the day the questionnaire is administered. 

### 2.6. Statistical Analysis

The information collected in this study was tabulated in an anonymised database designed specifically for this study. IBM SPSS Statistics software (Version 21, IBM SPSS, Chicago, IL, USA) was used for statistical analysis.

Data were presented as mean and standard deviation (SD) and as percentage. After analysing the normality of each of the study variables using the Kolmogorov-Smirnov test and verifying that the data did not follow a normal distribution, Spearman’s correlation coefficient was calculated between the SF12v2, FKQ and EQ-5D-5L questionnaires. The Bonferroni correction was applied based on the formula α* = α/n − 1 [27], where α* is the corrected value at which the null hypothesis should be rejected and n is the number of hypothesis pairs. Therefore, the alpha significance level was set at 0.001 for multiple comparisons between the FKQ and the SF12. To define the type of correlation between the variables, Cohen’s ratio [28] was used: 0.10 to 0.29 small correlation; 0.30 to 0.59 moderate correlation; 0.6 to 0.79 high, and ≥0.8 excellent. To analyse the FKQ domains associated with a greater impact of the quality of life of FM (EQ 5D-5L), a multivariate linear regression model was designed, in which the dependent variable was the score on the EQ 5D-5L and the independent variables were those that were significant in the bivariate analysis. Statistical significance was defined for *p* < 0.05.

## 3. Results

The total number of participants was 121 with a mean age of 55.06 (±9.93). In this way, Table 1 shows the characterization of the participants.

Table 2 shows that most of the participants had primary education (47.8%), followed by secondary education (29.8%).

Table 3 shows the percentages of participants at each level according to the scores obtained in each domain of the FKQ and for the total of score. For this purpose, the following score ranges were established: less than 50% of the maximum possible score (low knowledge), from 50 to 75% (medium knowledge) and more than 75% (high knowledge). In this table, it can be seen that for all the domains and for the total of the questionnaire, the highest percentages are in the range 50–75%, followed by the range +75% regarding the general and physical activity domains, as well as the total of score.

The response percentages related to the total sample are shown in Table 4. These percentages are expressed for each response option within its corresponding item. It can be seen that there are several response options that are not correct, but with a high percentage of responses from the participants (item 1 option b = 43%; item 8 option d = 49% and option e = 63%; item 11 option b = 44%), as well as some correct responses with a low number of responses (item 17 option b = 13%).

Table 5 shows the means in the different dimensions of the SF12v2 of the participants, compared to normal values for healthy women between 55–64 [29]. We can highlight that the values of the study sample are below normal values for healthy women between 55–64 in Spain, especially in the dimensions of physical function, body pain, general health vitality and physical health index [29].

The association between the FKQ and the SF12v2 is shown in Table 6. It can be observed that there is not significative correlation between FKQ and the SF12v2. 

Before performing the Bonferroni correction, a multivariate analysis was performed on those that were significant, and the results obtained were show in Table 7.

Table 8 shows the multivariate analysis revealed that the physical activity domain of FKQ was the only domain that were associated with a higher HQRoL.

Physical activity habits are shown in Table 9, where it is observed that with regard to the hours/week of practice, the highest percentages are found in “between 1 and 2 h” (31.4%) and “between 3 and 4 h” (24.4%). In relation to the distance walked daily, the highest percentages are found in “between 1 and 2 km” (46.3%) and “between 3 and 5 km” (21.5%).

Finally, Table 10 shows the association between knowledge measured by the FKQ and physical activity habits, showing that there is no correlation between the level of knowledge of the disease and the hours of physical exercise, nor in relation to the distance spent walking per week.

## 4. Discussion

To our knowledge, this is the first study on the assessment of knowledge of FM in women with FM in Extremadura. The main finding of the study was to determine that the level of knowledge about FM of patients in Extremadura was medium-high. As a secondary finding, we can highlight the existence of a weak direct correlation between knowledge about physical activity in FM and HRQoL, i.e., the greater the knowledge about physical activity, the greater the HRQoL. However, any association was found between knowledge of FM and HRQol evaluated by SF12v2. Furthermore, in accordance with the objectives of the study, no association was found between knowledge of physical activity in FM and the practice of physical activity.

Overall, the participants’ level of knowledge about FM was medium (49%) and high (41%), with the dimensions medication and energy being the ones where the highest number of participants had low knowledge (29% and 30% respectively). 

Specifically, for the general domain, in item 1, related to the causes of FM, it can be observed that there is a great diversity in the answers, with the correct options being chosen by only 56% and 58% of the participants, similar to the results reported by a similar study carried out by Moretti, et al. [30], which highlighted that 69% of their sample knew the cause of FM. In relation to the results obtained for this question (item 1), one of the most chosen options (43%) is that FM is due to physical trauma, so we can affirm that there is still a lack of knowledge about the cause among people with FM, in line with Alvarado Moreno and Oliva Arias [31], who highlight that the most deficient knowledge about FM is related to the origin and treatment. 

It can be observed that the medication domain is one of those in which it was found the greatest lack of knowledge, since the responses are very diverse (items 7, 8 and 9), something which is supported by other studies in which this domain has shown the lowest score [31,32]. Specifically, in item 8 it can be observed how 50% of the participants chose the option of “regular exercise and anti-inflammatory drugs” as correct, which could be due to the lack of guidance from their doctors about the suitability of the different treatments or drugs, since as highlighted by several studies such as Blotman, et al. [33], 20% of the doctors participating in their study took nonsteroidal anti-inflammatory drugs as suitable medication. Furthermore, the study conducted by Kianmehr, et al. [34] stated that 53.2% of the general practitioners participating in the study had low or very low levels of knowledge about the treatment of FM, and more specifically 52.1% of them also marked the use of nonsteroidal antinflammatory agents. In this line and at a general level, Ortiz, et al. [35] tated that the percentage of physicians with knowledge about “Drugs with proven usefulness” was slightly higher than half of the respondents (59.3%). 

In the domain of physical activity, the results show that in the question on exercise and body pain (item 10), a hit rate of 78% was found, in line with that reported by Moretti, et al. [30] (89% hit rate). However, Alvarado Moreno and Oliva Arias [31] found only a 29.2% accuracy rate, attributing this to the poor knowledge of physicians in their region about appropriate physical therapy and exercise-based treatments [35]. In relation to the importance of physical activity (item 11), we can find that there is a high response rate (44%) for “when the patient suffers pain, the best thing to do is to stay in bed” compared to the correct response “they should do physical exercise three times a week” (48%), far from what was reported by another study with 82% correct [30].

For the energy domain, in relation to item 17, we can say that there is a great lack of knowledge about protection in relation to energy, as 53% of the participants answered that they “did not know”, with only 13% selecting the correct answer (carrying the bags on the forearm instead of in the hands), similar to what reported Moretti et al. [30], where 27% of the participants did not know the correct answer. 

In relation to the participants’ HRQoL, the scores on the different dimensions of the SF12v2 of the participants are well below the normal values for healthy women aged 55–64 [29], especially in the dimensions of physical function, body pain, general health, vitality and physical health index, which highlights the HRQoL deficiencies of FM patients. 

Several studies claim that patients’ knowledge of the disease contributes to disease control [15,36]. Most of them use disease education within a treatment programme or multidisciplinary programme, resulting in improvements in both the impact of FM and HRQoL [36,37]. Certainly the evaluation of an FM education programme in isolation has been little studied, so further studies would be relevant to determine the level of influence or weight that knowledge of the disease could have in such multidisciplinary programmes, as the results obtained in this study reveal that greater knowledge of the disease, in this case about physical activity, could be associated with better HRQoL. 

Regarding the association between knowledge and physical activity practice, no association was found between the level of physical activity practice and the level of knowledge. In other words, it cannot be affirmed that those who do more physical activity have better knowledge of the disease, not even in the specific domain of physical activity. 

This study has several limitations, including the fact that the sample was based on women only. In addition, descriptive data such as weight and height were not taken, so the anthropometric characteristics of the sample cannot be observed. Furthermore, it was not possible to establish cause-effect relationships due to the cross-sectional nature of the present study, for which experimental studies would be necessary. 

This study shows that knowledge about FM in women with the pathology could influence some aspects of their HRQoL. Therefore, this possible improvements both at the level of the patient and the symptomatology, as well as at the level of the health service, if this would allow cost reduction, could be beneficial for patients as already being in other diseases such as diabetes [38,39,40] or arthritis [41,42], where there are many educational programmes aimed at different populations. In FM field this is still underdeveloped, so it would be very interesting to implement them. Furthermore, FM patients suffer a high lifetime rate of comorbid like migraine, irritable bowel syndrome, chronic fatigue syndrome, major depression, panic disorder, etc, therefore, it would be very interesting in future studies to know to what extent these comorbidities could influence both knowledge about the disease, as well as the relationship between knowledge and HRQOL. 

## 5. Conclusions

Based on the results of the present study, it could be concluded that the level of knowledge about FM of patients in Extremadura was medium-high and there is a direct relationship between knowledge about physical activity in FM patients and their HRQoL. However, no association was found between knowledge of physical activity in FM patients and their practice of physical activity.

## Figures and Tables

**Table 1:** Characterization of study patients.

	N	Means (SD)
Age (years)	121	55.06 (9.93)
Years of accurate diagnosis	118	4.94 (0.65)
Years suffering from generalized pain	114	15.21 (10.24)
Number of members of the family unit	106	2.71 (1.17)
Monthly household income (Euros)	94	528.37 (862.50)
Number of trigger points (0–18)	85	7.07 (5.32)
Degree of pain at the time of the questionnaire (0–100)	119	68.06 (17.68)

**Table 2:** Educational level of study patients.

	N (%)
Illiterate	1 (0.8)
No education but can read and write	9 (7.4)
Primary education	58 (47.9)
Secondary education	36 (29.8)
Graduate	14 (9.9)
Other	3 (2.5)

**Table 3:** Percentage of participants at each level of knowledge according to the scores obtained for each domain of the FKQ.

	FKQ														
	General			Medication			Physical Activity			Energy			Total		
	Range	n	%	Range	n	%	Range	n	%	Range	n	%	Range	n	%
−50%	0–4	11	10	0–2	35	29	0–2	18	15	0–2	37	30	0–11	12	10
50–75%	5–7	62	51	3–4	66	55	3–4	67	55	3	71	59	12–17	59	49
+75%	8–9	48	39	5–6	20	16	5	36	30	4	13	11	18–23	50	41

Range: range of scores for the levels established according to each domain. FKQ: Fibromyalgia Knowledge Questionnaire.

**Table 4:** Percentage of responses for each possible option within each item in the FKQ.

Response Option		a	b	c	d	e	f
FKQ Domains	Item	(%)	(%)	(%)	(%)	(%)	(%)
**General**	1	13	43	56 *	7	58*	11
	2	5	77 *	15	3	94*	5
	3	3	0	0	97 *	1	
	4	75 *	7	12	13	62 *	10
	5	6	82 *	53*	2	31	
**Medication**	6	73 *	4	17	4	2	7
	7	2	41 *	17	13	28	
	8	4	50	3	49 *	63 *	12
	9	26	62 *	44 *	17	27	
**Physical Activity**	10	7	3	2	78 *	10	
	11	0	44	48 *	0	9	
	12	92 *	1	1	0	6	
	13	74 *	7	4	77 *	15	
**Energy**	14	4	0	93 *	2	2	
	15	2	95 *	83 *	3	5	
	16	75 *	1	15	0	14	
	17	11	13 *	2	23	53	
	18	6	3	0	10	72 *	10

* Correct answer for the corresponding item.

**Table 5:** SF12v2 averages.

	Means (SD) (n = 121)	Means (SD) (n = 188)
**Physical Function**	30.55 (8.51)	45.4 (13.4)
**Physical Rol**	34.63 (7.68)	45.4 (13.8)
**Body Pain**	30.99 (9.07)	46.6 (6.5)
**General Health**	27.74 (8.48)	37.4 (11.0)
**Vitality**	37.93 (9.93)	52.0 (12.7)
**Social Rol**	36.70 (11.58)	47.6 (13.5)
**Emotional Rol**	37.04(10.69)	45.01 (14.6)
**Mental Health**	40.86 (5.63)	47.3 (14.2)
**Mental Health Index**	42.08 (8.21)	42.9 (13.1)
**Physical Health Index**	29.02 (8.37)	48.9 (14.1)

**Table 6:** Association between FKQ and SF12v2.

	FKQ				
	General	Medication	Physical Activity	Energy	Total
**Physical Function**	0.074	0.019	0.161	−0.051	0.087
**Physical Rol**	−0.025	0.009	0.171	−0.021	0.015
**Body Pain**	0.023	0.051	0.203	0.057	0.095
**General Health**	0.109	−0.051	0.154	−0.008	0.058
**Vitality**	−0.033	−0.104	0.024	−0.155	−0.065
**Social Rol**	0.028	0.112	0.143	0.043	0.017
**Emotional Rol**	0.156	0.130	0.207	0.006	0.209
**Mental Health**	0.130	0.166	0.135	0.049	0.169
**Mental Health Index**	0.086	0.014	0.133	−0.009	0.138
**Physical Health Index**	−0.012	−0.050	0.105	−0.059	−0.008

FKQ: Fibromyalgia Knowledge Questionnaire.

**Table 7:** Domain of FKQ that is associated with the body pain.

Body Pain (R^2^: 0.067)	*B*	*t*	*p* Value
Constant	12.60	1.566	0.120
FKQ Physical Activity	5.850	2.932	0.004

**Table 8:** Domain of FKQ that is associated with a greater HRQoL.

EQ 5D-5L (R^2^: 0.078)	*B*	*t*	*p* Value
Constant	0.165	1.986	0.049
FKQ Physical Activity	0.066	3.180	0.002

**Table 9:** Sports practice habits.

		N (%) 121 (100)
**Hours (per week)**	None	21 (17.4)
	Less than 1 h	13 (10.7)
	Less than 1 and 2 h	38 (31.4)
	Less than 3 and 4 h	30 (24.4)
	Less than 5 and 8 h	16 (13.2)
	Less than 9 and 14 h	2 (1.7)
	More than 21	1 (0.8)
**Distance (daily)**	Does not walk	19 (15.6)
	Less than 1 km	16 (13.)
	Between 1 and 2 km	56 (46.3)
	Between 3 and 5 km	26 (21.5)
	Between 6 and 9 km	2 (1.7)
	More than 9 km	2 (1.7)

Hours: How many hours per week do you exercise? Distance: approximately indicate how far you walk daily.

**Table 10:** Correlation between FKQ and physical exercise.

	FKQ				
	General	Medication	Physical Activity	Energy	Total
**Hours**	0.020	0.107	0.070	0.181	0.102
**Distance**	0.174	0.092	0.004	0.117	0.134

FKQ: Fibromyalgia Knowledge Questionnaire.

## Data Availability

The datasets used during the current study are available from the corresponding author on reasonable request.
